# Treatment completion rates and adverse effects of three months of once-weekly isoniazid plus rifapentine for latent tuberculosis infection

**DOI:** 10.36416/1806-3756/e20250234

**Published:** 2025-10-01

**Authors:** Tiene Heidy Maoski, Giovana Rodrigues Pereira, André Kulzer Santos, Raimunda Sinthia Lima de Braga, Marina Scheffer de Souza, Gean Souza Ramos, Allanamara Pereira Marinho, Renata Ullmann de Brito Neves, Denise Rossato Silva

**Affiliations:** 1. Programa de Pós-Graduação em Ciências Pneumológicas, Universidade Federal do Rio Grande do Sul - UFRGS - Porto Alegre (RS) Brasil.; 2. Laboratório Municipal de Alvorada, Alvorada (RS) Brasil.; 3. Faculdade de Medicina, Universidade Federal do Rio Grande do Sul - UFRGS - Porto Alegre (RS) Brasil.; 4. Centro de Referência para Tratamento de Tuberculose Navegantes, Prefeitura Municipal de Porto Alegre, Porto Alegre (RS) Brasil.; 5. Serviço de Pneumologia, Hospital Nossa Senhora da Conceição - HNSC - Porto Alegre (RS) Brasil.; 6. Serviço de Pneumologia, Hospital de Clínicas de Porto Alegre - HCPA - Porto Alegre (RS) Brasil.

**Keywords:** Tuberculosis, pulmonary, Latent tuberculosis/prevention & control, Mycobacterium tuberculosis

## Abstract

**Objective:**

Preventive treatment of active tuberculosis is one of the main strategies for reducing the incidence of tuberculosis. We sought to evaluate the rates of latent tuberculosis infection (LTBI) treatment completion with three months of once-weekly isoniazid plus rifapentine (3HP) and compare them with those for six to nine months of daily isoniazid (6H/9H).

**Methods:**

This was a retrospective cross-sectional study. Consecutive patients undergoing LTBI treatment with 3HP or 6H/9H were included in the study. Treatment completion rates and adverse effects were analyzed.

**Results:**

A total of 226 patients were included in the study: 113 in the 3HP group and 113 in the 6H/9H group. The frequency of adverse effects was not significantly different between the 3HP and 6H/9H groups. The 3HP group had a higher treatment completion rate (93.8%) than did the 6H/9H group (84.1%), the difference being significant.

**Conclusions:**

The rates of LTBI treatment completion appear to be higher with 3HP than with 6H/9H. Health care professionals should be vigilant in managing adverse effects to further maximize LTBI treatment completion.

## INTRODUCTION

Most people infected with *Mycobacterium tuberculosis* are asymptomatic, a condition known as latent tuberculosis infection (LTBI). According to the WHO, approximately 2-3 billion people worldwide are infected with *M*. *tuberculosis*, and 5-15% will progress from LTBI to active symptomatic disease during their lifetime. Reactivation of LTBI accounts for a large proportion of the incidence of active tuberculosis, making diagnosis and treatment crucial, especially in high-risk groups.^1^ ^,^ ^2^

Preventive treatment of active tuberculosis is one of the main strategies for reducing the incidence of tuberculosis. There are several regimens for treating LTBI. However, since 2021, the Brazilian National Ministry of Health has recommended three months of once-weekly isoniazid plus rifapentine (3HP) as the first choice of treatment. There are at least four randomized clinical trials^3^ ^-^ ^6^ demonstrating that the 3HP regimen is as effective as isoniazid monotherapy, with higher treatment completion rates. The 3HP regimen is used once a week for three months, for a total of 12 doses. Its efficacy and toxicity are similar to those of six months of daily isoniazid (6H), the main advantage being reduced treatment time.^7^

Despite the advantages of the 3HP regimen, some patients may experience influenza-like symptoms or hypersensitivity reactions. Although these are less common than symptoms such as pruritus, rash, nausea, and vomiting, they remain a cause for concern among patients and physicians alike regarding the use of the 3HP regimen.^8^ One study demonstrated that patients using 3HP are at an increased risk of adverse effects leading to treatment discontinuation.^9^ In Brazil, there have been no studies evaluating 3HP treatment completion rates or the profile of adverse events associated with the 3HP regimen. Therefore, the objective of the present study was to evaluate the rates of LTBI treatment completion with 3HP and compare them with those of LTBI treatment completion with 6H or nine months of daily isoniazid (9H). 

## METHODS

### Study design and setting

This was a retrospective cross-sectional study conducted in the city of Alvorada, in southern Brazil. The study was approved by the local institutional review board on October 11, 2023 (Protocol no. 6.423.958). 

### Patients and data collection

Consecutive patients undergoing LTBI treatment with 3HP or 6H/9H were included in the study. We included all of the patients who initiated treatment with the 3HP regimen and the same number of patients initiating treatment with the 6H/9H regimen. The exclusion criterion was having undergone LTBI treatment with four months of rifampin. We collected the following data: demographic data (including age, sex, and race); smoking status; alcohol abuse; drug use; comorbidities; LTBI treatment regimen; adverse effects of LTBI treatment; and treatment outcome (completion or abandonment). 

### Statistical analysis

Data analysis was performed with the IBM SPSS Statistics software package for Windows, version 22.0 (IBM Corporation, Armonk, NY, USA). Data were presented as number of cases, mean ± standard deviation, or median [interquartile range]. Categorical comparisons were performed by using the chi-square test with Yates’ correction (when appropriate) or Fisher’s exact test. Continuous variables were compared by using the *t*-test or the Wilcoxon test. A two-sided value of p < 0.05 was considered significant for all analyses. 

In order to calculate the sample size, we used treatment completion rates of approximately 90% for the 3HP regimen and 70% for the 6H/9H regimen.^5^ ^,^ ^10^ Thus, considering a 95% confidence interval and a study power of 80%, we calculated a sample size of 54 patients per group. 

## RESULTS

A total of 226 patients were included in the present study. Of those, 113 were included in the 3HP group and 113 were included in the 6H/9H group. The characteristics of the study population, by treatment regimen, are shown in Table 1. The mean age was 41.2 ± 18.7 years in the 3HP group and 40.3 ± 18.6 years in the 6H/9H group (p = 0.738). In the 3HP and 6H/9H groups, 53.1% and 58.4% were male, respectively. Current smoking, alcohol abuse, and drug use were reported in the 6H/9H group only, by 2.7%, 2.7%, and 1.8%, respectively. The prevalence of comorbidities was not significantly different between the 3HP and 6H/9H groups (23.0% vs. 19.5%; p = 0.626). HIV infection was present in 7 (6.2%) of the patients in the 3HP group and in 9 (8.0%) of those in the 6H/9H group (p = 0.795). 

**Table 1:** Comparison of patients with latent tuberculosis infection treated with three months of once-weekly isoniazid plus rifapentine or six to nine months of daily isoniazid.^a^

Characteristic	Group		p
	3HP	6H/9H	
	(n = 113)	(n = 113)	
Age, years	41.2 ± 18.7	40.3 ± 18.6	0.738
Male	60 (53.1)	66 (58.4)	0.503
White	46 (40.7)	49 (43.4)	0.788
Current smoking	0	3 (2.7)	0.247
Alcohol abuse	0	3 (2.7)	0.247
Drug use	0	2 (1.8)	0.498
Comorbidities	26 (23.0)	22 (19.5)	0.626
HIV infection	7 (6.2)	9 (8.0)	0.795
Adverse effects	29 (25.7)	24 (21.2)	0.530
Nausea/vomiting	16 (55.2)	22 (91.7)	0.009
Abdominal pain	14 (48.3)	2 (8.3)	0.004
Headache	9 (31.0)	0	0.003
Default from treatment	7 (6.2)	18 (15.9)	0.034

3HP: three months of once-weekly isoniazid plus rifapentine; and 6H/9H: six to nine months of daily isoniazid. ^a^Data presented as n (%) or mean ± SD.

The frequency of adverse effects was not significantly different between the 3HP and 6H/9H groups. However, nausea/vomiting was more common in the 6H/9H group (in 22 [91.7%]) than in the 3HP group (in 16 [55.2%]; p = 0.009). In contrast, abdominal pain and headache were more common in the 3HP group than in the 6H/9H group (p = 0.004 and p = 0.003, respectively). Seven (6.2%) of the patients in the 3HP group and 18 (15.9%) of those in the 6H/9H group defaulted from treatment, the difference being statistically significant (p = 0.034). The 3HP group had a higher treatment completion rate (93.8%) than did the 6H/9H group (84.1%). 

## DISCUSSION

In this cross-sectional study, we found that patients using the 3HP regimen had a higher treatment completion rate (93.8%) than did those using the 6H/9H regimen (84.1%). In addition, nausea/vomiting was more common in the 6H/9H group, whereas abdominal pain and headache were more common in the 3HP group. 

Treating LTBI is crucial to prevent it from developing into active tuberculosis, LTBI treatment being an important component of tuberculosis control and elimination.^11^ Until recently, the treatment of choice for LTBI in Brazil was six to nine months of daily isoniazid. Problems such as hepatotoxicity, as well as the long duration of treatment, contribute to low rates of treatment completion with 6H/9H, ranging from 30% to 64%.^5^ Thus, shorter treatment regimens for LTBI are very welcome. In this sense, studies^5^ ^,^ ^6^ have shown that the 3HP regimen has a higher treatment completion rate than does the 9H regimen, as well as having similar effectiveness. In an open-label, randomized noninferiority trial, the rates of treatment completion were 82.1% in the 3HP group and 69.0% in the 9H group.^5^ In another study, treatment completion was higher with 3HP (89%) than with 9H (64%).^6^ In our study, treatment completion rates were higher than the aforementioned rates in both groups, although ours was a pragmatic study without the ideal conditions of a clinical trial. 

Nausea and vomiting were more common in the 6H/9H group than in the 3HP group in the present study. It is well known that gastrointestinal effects constitute the most common group of reactions and can be attributed to any drug. A major concern with isoniazid is the possibility of hepatitis. During treatment, as many as 10% to 20% of patients treated with isoniazid have mild, asymptomatic elevations in liver enzymes, but hepatitis is rare. Nausea and vomiting are also symptoms of hepatitis, in addition to fatigue, poor appetite, jaundice, and abdominal pain.^12^ In our study, we did not have any cases of hepatitis. 

Abdominal pain and headache were more common among patients in the 3HP group than among those in the 6H/9H group. As mentioned above, abdominal pain is part of the set of gastrointestinal symptoms that are the most common group of symptoms and are related to several drugs. Headache is also a common side effect, being usually mild and transient, and most patients are able to complete treatment without interruption. In a study analyzing data on symptoms in 1,002 participants receiving 3HP, the most common symptom was headache (in 29.4%).^13^

Our study has some limitations. First, we recruited patients from a single setting; however, we do not think that this is a limitation for generalizing the results. Second, the study was retrospective, being based on patient medical records, which may compromise the completeness of the data. Finally, adverse effects were not graded, because they were collected in the context of clinical practice rather than for a clinical trial. Despite these concerns, this was the first study in Brazil to evaluate the use of the 3HP regimen in pragmatic conditions. 

In conclusion, the rates of LTBI treatment completion with 3HP are higher than those with 6H/9H. Health care professionals should be vigilant in managing adverse effects to further maximize LTBI treatment completion. 
